# Pregnancy Achieved One Month After Laparoscopic Myomectomy With a Favorable Delivery Outcome: A Case Report

**DOI:** 10.7759/cureus.93295

**Published:** 2025-09-26

**Authors:** Munetoshi Akazawa, Kazunori Hashimoto

**Affiliations:** 1 Obstetrics and Gynecology, Tokyo Women’s Medical University Medical Center East, Tokyo, JPN; 2 Obstetrics and Gynecology, Tokyo Women's Medical University, Tokyo, JPN

**Keywords:** cesarean section, laparoscopic myomectomy, perinatal management, pregnancy, uterine rapture

## Abstract

The optimal interval between myomectomy and pregnancy is often considered to be 3-6 months after laparoscopic myomectomy, as it is necessary to wait for the uterine wall to heal. However, no guidelines from any country were found to support this specific timeframe explicitly. Given the current trend of older maternal age, the contraceptive period following myomectomy can lead to reduced pregnancy success rates. Further consideration of the permitted pregnancy period after myomectomy is warranted. Against this background, we present a case of pregnancy occurring one month after myomectomy in a 40-year-old woman. She underwent laparoscopic myomectomy for multiple uterine fibroids and achieved spontaneous pregnancy with a menstrual period of one month after surgery. She delivered a 2,692-gram infant by scheduled cesarean section at 37 weeks of gestation, with no notable postoperative complications. We reviewed the literature regarding the permitted pregnancy period following laparoscopic myomectomy.

## Introduction

Uterine fibroids are the most common tumors, which occur in over 70% of women by the onset of menopause [1]. Fibroids can cause heavy menstrual bleeding, bladder or bowel dysfunction, and adversely affect fertility and obstetric outcomes [1]. For the clinical complaints or improvement of obstetrical outcome, myomectomy is performed in women who desire to receive curative treatment. Myomectomy is the gold standard fertility-sparing treatment for uterine fibroids and is considered to be associated with better pregnancy outcomes than other fertility-preserving treatments such as ulipristal acetate, uterine artery embolization, and ablation [2].

Although myomectomy is widely performed clinically, the optimal interval between myomectomy and pregnancy is unclear, and specific guidelines have not been issued [3]. In pregnancies following myomectomy, uterine rupture is the primary perinatal complication of pregnancy [4]. Uterine rupture is defined as a complete tear of the uterine wall, which is an obstetric emergency, resulting in high morbidity and mortality rates in both the mother and fetus [4]. Thus, a 3-6 month waiting period has been recommended to allow the uterus to heal for pregnancy [3].

However, with the current trend of increasing pregnancy rates in older women, a longer waiting period may lead to a decline in ovarian function and potential fibroid recurrence. Therefore, delaying pregnancy initiation could result in a loss of pregnancy opportunities. In our case, a spontaneous pregnancy occurred during the menstrual cycle one month after myomectomy. The pregnancy progressed without complications, and a healthy infant was delivered at 37 weeks via a routinely scheduled cesarean section. The purpose of reviewing this case was to examine the turning points in cases of early pregnancy following laparoscopic myomectomy through a literature review, while also considering the multifaceted management approach for such early pregnancies after myomectomy.

## Case presentation

**Figure 1:**
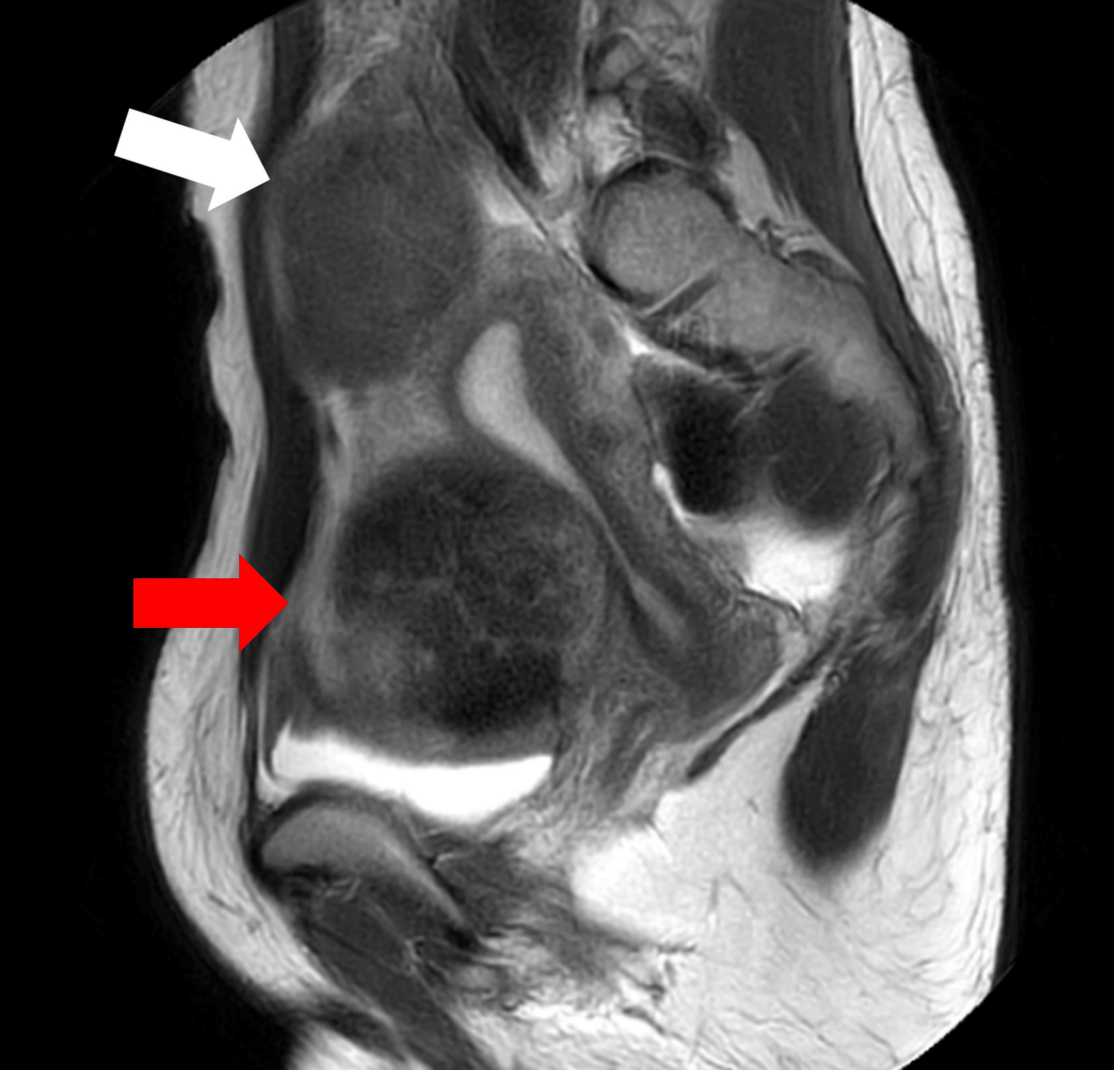
Pelvic MRI examination Pelvic MRI image showed a 6cm fibroid at the fundus (white arrow) and 9cm fibroid on the left side which distorted the uterine cavity (red arrow).

A 39-year-old patient (gravida 0, para 0) presented to primary care complaining of a sensation of a mass in the lower abdomen and pain during menstruation. She had no prior medical history and no history of infertility treatment. Examination revealed uterine fibroids measuring 9 cm and 6 cm. The patient was referred to our department for consultation. During further evaluation, pelvic magnetic resonance imaging (MRI) revealed a 69 × 55 mm fibroid at the fundus and a 93 × 85 mm fibroid on the left side of the anterior uterine wall that distorted the uterine cavity (Figure 1).

**Figure 2:**
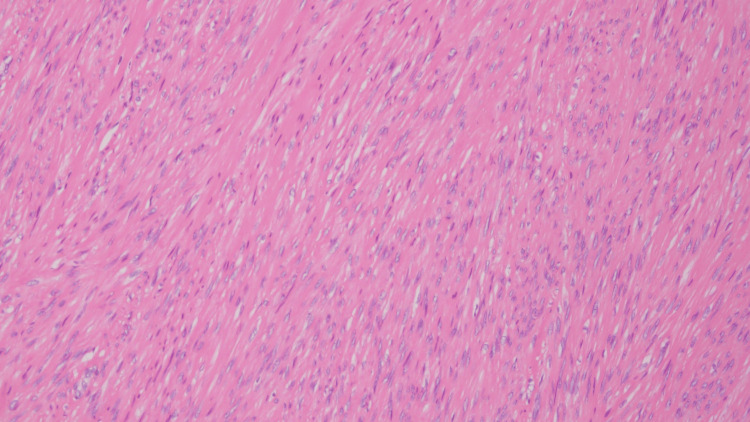
Histopathological image of uterine myoma Smooth muscle-like spindle-shaped tumor cells are arranged in bundles.

The fibroid at the fundus of the uterus was defined as Group 4 according to the 2011 FIGO (the International Federation of Gynecology and Obstetrics) classification, while the left fibroid was defined as Group 3 [5]. The patient opted for laparoscopic myomectomy. During surgery, the two fibroids were removed without entering the uterine cavity using a harmonic energy device. For the largest posterior fundal fibroid, repair was performed using a barbed suture material, with two layers of continuous sutures in the myometrium and one layer of continuous sutures in the serosal layer, for a total of three layers. The 6 cm fibroids were similarly repaired using three layers of sutures. Subsequently, all fibroids were dissected into small pieces and excised via a small laparotomy incision. The pathological diagnosis was a uterine myoma (Figure 2).

**Figure 3:**
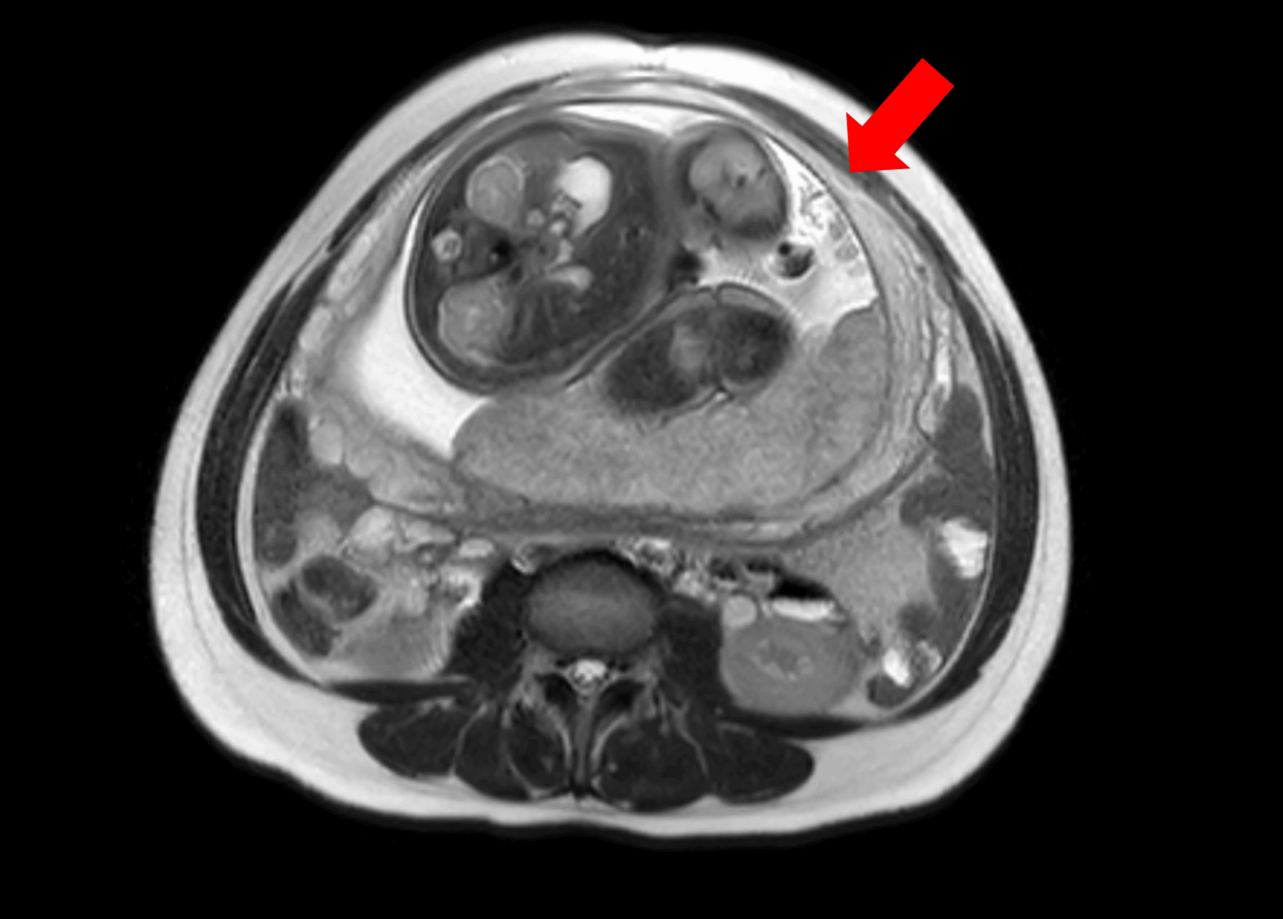
Pelvic MRI examination during pregnancy MRI images during pregnancy suggested no muscle thinning on the left side of the anterior wall (red arrow). There was no obvious thinning of the myoma suggestive of uterine rupture, so delivery at preterm was considered unnecessary.

The patient had recovered well at the one-month postoperative follow-up. We explained the need for contraception for three months postoperatively and scheduled a three-month follow-up, but the patient did not attend. Subsequently, she visited primary care facilities three months after surgery, complaining of amenorrhea. She was diagnosed with an intrauterine pregnancy using transvaginal ultrasonography. The crown-rump length (CRL) of the fetus was 23 mm (equivalent to 8 weeks of gestation). The placenta was located on the posterior wall of the uterus. Since postoperative amenorrhea persisted, and the date of the last menstrual period was unknown, the estimated delivery date was calculated based on the CRL. This estimated date suggested that the last menstrual period could have been one month following surgery. The patient was then referred to our department for perinatal management. Subsequently, she continued prenatal care at our hospital, including regular abdominal ultrasound and pelvic MRI examinations at 34 gestational weeks to assess the myometrial condition. The MRI imaging suggested no muscle thinning on the left side of the anterior wall (Figure 3).

Her BMI during pregnancy was 20.4, and she gained 9.3 kg during pregnancy. In line with national pregnancy guidelines, we recommended that gestational weight gain be determined by the patient’s BMI, with a target range of 10-13 kg. Additionally, we recommended a nutritionally balanced diet and moderate aerobic exercise that is not excessive. We strongly emphasized that early pregnancy following myomectomy carries a high risk of uterine rupture. We instructed the patient to contact the hospital immediately if symptoms such as abdominal pain or genital bleeding occurred during pregnancy. The patient did not experience these symptoms and did not present for any unscheduled prenatal visits.

Due to prior myomectomy, we planned a cesarean section at 37 gestational weeks, earlier than usual. The delivery proceeded without complications at 37 gestational weeks and 2 days, resulting in the birth of a healthy infant weighing 2692 g. The Apgar scores were 8 at 1 min and 9 and 5 min, respectively. Surgical findings revealed no macroscopic issues with the previous myomectomy incision site, and no thinning was observed. Both mother and infant had an uneventful postpartum course and were discharged on the 7th postpartum day. At the one-month postpartum follow-up, no abnormalities were found in either the mother or the infant, and outpatient care was discontinued.

## Discussion

Uterine rupture is the primary perinatal complication of pregnancy after myomectomy [4]. It is defined as a complete tear of the uterine wall, accompanied by rupture of the overlying visceral peritoneum. The incidence of uterine rupture after myomectomy has been reported to be 0.9% (0.3-1.1%) in women who underwent myomectomy [4]. Uterine rupture, which is an obstetric emergency, results in high morbidity and mortality rates in both the mother and fetus. Risk factors such as the surgical approach, type of hemostasis, suturing technique during myomectomy, and characteristics of the excised fibroids have been investigated in the literature; however, whether the interval since surgery is a risk factor for uterine rupture remains inconclusive. The rate of uterine rupture was reported to be highest during childbirth occurring within one year after myomectomy and decreased with increasing time since surgery [6]. This study reported a decline from 0.71% in the first year to 0.35% in the second year and 0.21% in the third year; however, the incidence within the first year was not further subdivided. Regarding the timing of uterine rupture during pregnancy, the mean gestational age at rupture was reported as 31.0 weeks (standard deviation 7.0 weeks) [3]. The earliest pregnancy week in which uterine rupture occurred was at 10 weeks of gestation [3].

No guidelines have been issued by any gynecological association regarding the optimal interval between myomectomy and pregnancy, or the minimum interval before attempting pregnancy postoperatively. Regarding the pregnancy period actually explained by healthcare providers, one-third of respondents reported recommendations of waiting 3-6 months, and more than a third between 6 and 12 months before attempting pregnancy [3]. In cases in which pregnancy occurred, the average time from surgery to conception was 17.6 months, with a median of 13.3 months. Delaying pregnancy after myomectomy increases the risk of uterine recurrence and further fibroid development owing to aging, potentially leading to infertility [7]. By evaluating the clinical factors associated with pregnancy probability after myomectomy using multivariate analysis, several factors were associated with an increased likelihood of postoperative pregnancy, including younger age at surgery, fibroid size, fibroid location (intramural), and laparoscopic surgery. Younger patients who underwent surgery had a higher likelihood of pregnancy [8]. In our case, although the patient was not young, the large fibroid size (over 6 cm in diameter) and its partial submucosal location, combined with the laparoscopic approach, were considered factors contributing to the improved pregnancy rate.

In the individual case reports, no reports of pregnancy occurring within one or two months postoperatively were found via a PubMed search. In a systematic review encompassing multiple case series from single or multiple institutions that analyzed the pregnancy after myomectomy, nine papers identified cases of pregnancy where the shortest interval from myomectomy to pregnancy was 1 month [3]. Although the clinical course of these cases is unclear, pregnancy is thought to occur immediately after myomectomy, although this is rare. Several case reports have described myomectomy performed during early pregnancy. A case of an unexpected pregnancy at the time of robotic myomectomy was reported. In this case, the patient underwent robot-assisted laparoscopic myomectomy during the gestational period of four weeks despite a negative preoperative pregnancy test [9]. After confirming the pregnancy, the patient wished to continue the pregnancy and delivered a healthy baby at 38 weeks. Additionally, two cases are recognized where emergent myomectomy was performed at an early stage of pregnancy. One was a case of a large, asymptomatic subserosal uterine fibroid diagnosed during pregnancy. Open myomectomy at 14 weeks of gestation successfully preserves fetal viability. At term, a male infant weighing 3 kg was delivered vaginally [10]. The other case was an exploratory laparotomy in a patient presenting with severe epigastric discomfort at 19 weeks of gestation who was diagnosed with a pregnancy-associated ovarian tumor. This revealed a 32 cm degenerated subserosal fibroid coexisting with an intrauterine pregnancy [11]. After myomectomy, the pregnancy progressed smoothly, and a female infant was delivered vaginally at 38 weeks of gestation. Regarding notable management during pregnancy for uterine rupture, in one case among three cases, an MRI examination was performed before delivery to assess myometrial thinning.

In our case, we performed laparoscopic myomectomy and achieved spontaneous pregnancy with the first menstruation one month after surgery, resulting in a successful cesarean section at 37 weeks. The patient was 39 years old at the initial consultation, but had turned 40 years old by the time of her surgical admission two months later. For patients around 40 years of age, a six-month pregnancy waiting period raises concerns about a potential decline in pregnancy rates. Although the pregnancy waiting period is currently set within a broad range of 3-6 months, more evidence-based and guideline-supported durations are clinically needed. Continued accumulation of evidence and establishment of a more scientifically grounded timeframe for pregnancy are required. Regarding pregnancy management, evaluation of the myometrium integrity via MRI in the second trimester and performing a cesarean section at 37 weeks of gestation may have reduced the risk of uterine rupture. In our report, MRI was performed at 36 weeks of gestation; however, since the median gestational age at uterine rupture occurrence was 31 weeks, MRI during the second trimester would have been preferable. Further consideration is needed regarding the appropriate pregnancy waiting period after myomectomy and the perinatal management of early pregnancy following myomectomy.

## Conclusions

The optimal interval between myomectomy and pregnancy is generally recommended to be 3-6 months after laparoscopic myomectomy to allow for sufficient healing of the uterine wall. However, no guideline explicitly supports a specific timeframe. For women undergoing myomectomy in their late 30s, as in the present case, the timing of potential pregnancy after myomectomy is a significant concern. Continued accumulation of evidence and establishment of a more scientifically grounded timeframe for pregnancy are required.
